# Extracting Common Mode Errors of Regional GNSS Position Time Series in the Presence of Missing Data by Variational Bayesian Principal Component Analysis

**DOI:** 10.3390/s20082298

**Published:** 2020-04-17

**Authors:** Wudong Li, Weiping Jiang, Zhao Li, Hua Chen, Qusen Chen, Jian Wang, Guangbin Zhu

**Affiliations:** 1 School of Geodesy and Geomatics, Wuhan University, 129 Luoyu Road, Wuhan 430079, China; liwudong@whu.edu.cn (W.L.); wpjiang@whu.edu.cn (W.J.); ttchen@whu.edu.cn (H.C.); winner@whu.edu.cn (J.W.); 2 GNSS Research Center, Wuhan University, Wuhan 430079, China; chenqs@whu.edu.cn; 3 Department of Land Surveying and Geo-Informatics, The Hong Kong Polytechnic University, 181 Chatham Road South, Hung Hom, Kowloon 999077, Hong Kong, China; 4 Key Laboratory of Earth Observation and Geospatial Information Science, Beijing 100039, China; sasmac_zgb@163.com; 5 Land Satellite Remote Sensing Application Center, Beijing 100048, China

**Keywords:** common mode error, variational Bayesian principal component analysis, GNSS position time series, missing data

## Abstract

Removal of the common mode error (CME) is very important for the investigation of global navigation satellite systems’ (GNSS) error and the estimation of an accurate GNSS velocity field for geodynamic applications. The commonly used spatiotemporal filtering methods normally process the evenly spaced time series without missing data. In this article, we present the variational Bayesian principal component analysis (VBPCA) to estimate and extract CME from the incomplete GNSS position time series. The VBPCA method can naturally handle missing data in the Bayesian framework and utilizes the variational expectation-maximization iterative algorithm to search each principal subspace. Moreover, it could automatically select the optimal number of principal components for data reconstruction and avoid the overfitting problem. To evaluate the performance of the VBPCA algorithm for extracting CME, 44 continuous GNSS stations located in Southern California were selected. Compared to previous approaches, VBPCA could achieve better performance with lower CME relative errors when more missing data exists. Since the first principal component (PC) extracted by VBPCA is remarkably larger than the other components, and its corresponding spatial response presents nearly uniform distribution, we only use the first PC and its eigenvector to reconstruct the CME for each station. After filtering out CME, the interstation correlation coefficients are significantly reduced from 0.43, 0.46, and 0.38 to 0.11, 0.10, and 0.08, for the north, east, and up (NEU) components, respectively. The root mean square (RMS) values of the residual time series and the colored noise amplitudes for the NEU components are also greatly suppressed, with average reductions of 27.11%, 28.15%, and 23.28% for the former, and 49.90%, 54.56%, and 49.75% for the latter. Moreover, the velocity estimates are more reliable and precise after removing CME, with average uncertainty reductions of 51.95%, 57.31%, and 49.92% for the NEU components, respectively. All these results indicate that the VBPCA method is an alternative and efficient way to extract CME from regional GNSS position time series in the presence of missing data. Further work is still required to consider the effect of formal errors on the CME extraction during the VBPCA implementation.

## 1. Introduction

In the last three decades, global navigation satellite system (GNSS) technology has provided abundant, high-accuracy position information for the Earth, which allows researchers to investigate many types of geophysical phenomena, such as geocenter motion [1,2], crustal deformation [3,4], seismic monitoring [5,6], and glacial isostatic adjustment [7,8]. However, the GNSS observations contain not only site-specific temporal noise, which is well quantified and described as a combination of white noise (WN) and power-law noises (PLN) using the maximum likelihood estimation (MLE) method [9], but also spatial correlation error known as the common mode error (CME) [10]. Interference between temporal noise and spatial correlation error would bias the station velocity estimation, increase velocity uncertainty, and even lead to incorrect geophysical interpretations; thus, it is highly recommended to mitigate the interference with some effective filtering techniques to enhance the GNSS signal-to-noise ratio.

For regional GNSS networks, strong CME, coming from the reference frame error, mis-modeling of satellite orbits and clocks, large-scale environmental effects, etc., presents in the position time series [11]. This makes it difficult to discern weak and transient tectonic signals, such as slow slip events and aseismic episodic tremor, from GNSS data. Until now, many methods have been proposed to filter out the CME, and Table 1 shows the overview of each mathematical method. The stacking approach was originally introduced to detect the seismic displacements [12]. After that, a weighted stacking method that takes individual position error into account was proposed [13]. This kind of stacking approach is based on the assumption of an equivalent CME for all stations; thus, it is not suitable for larger scale networks; e.g., continental networks, wherein CME exhibits inhomogeneous behavior. To overcome this restriction, Márquez-Azúa et al. took the length of the coordinate time series and site distance as weights to estimate the daily CME, and then removed it from the original GNSS time series to study the large-scale deformation of Mexico [14]. Tian et al. used the distances between neighboring sites and interstation correlations as a weighting scheme to extract the spatially correlated transients [15,16]. Nevertheless, the aforementioned methods are unable to identify stations with strong local effects that could affect the CME detection, and the suitable weights for all stations at each epoch still need to be investigated.

Another way to remove the CME is to transform the solutions into a regional reference frame [17,18]. In this way, the stations located at the edge of the region may exhibit distorted signals or some remnant CME [16]. To suppress the CME, more reliable, statistical signal decomposition techniques, such as principal component analysis (PCA) [19,20,21], multi-channel singular spectrum analysis (MSSA) [22,23], and independent component analysis (ICA) [24,25], have been introduced into GNSS time series analyses. Based on an assumed inhomogeneous distribution of CME and a more rigorous mathematical framework, these methods, in particular the PCA, have been widely used to eliminate CME from regional network. Nevertheless, the traditional PCA method normally requires a complete set of observations, indicating that interpolation should be implemented to recover the missing data beforehand, which may cause deviations in the extracted CME. Because of the limitation, Shen et al. [26] successfully developed an improved principal component analysis (IPCA) to extract CME from the discontinuous time series of 27 permanent GNSS stations in China. Then Li et al. [27,28] extended this approach by considering the formal errors as weights to extract CME. However, the IPCA approach is incapable of handling the condition that any two time series of a regional network have no, or only a few common epochs. Very recently, the probabilistic principal component analysis (PPCA) method was first introduced by Gruszczynski et al. [29] to filter out CME from time series with few common observational epochs, yet the PPCA method is sometimes sensitive to the initial parameters and prone to face the over-fitting problem due to the usage of maximum likelihood criterion to update model parameters [30].

To compensate for the above shortcomings, an alternative method called variational Bayesian principal component analysis (VBPCA) came into being [31]. The VBPCA method, as an extension of PPCA, imposed a priori distributions on the model parameters and estimated the hyperparameters by maximizing the evidence of observed signals instead of likelihood in the maximum-likelihood estimation paradigm. This property makes it more resistant against the overfitting problem in comparison with PPCA method. Moreover, the VBPCA method can recover the missing values in GNSS time series more conveniently, since it offers automatic dimensionality selection during the Bayesian inference procedure [30], while for other methods, i.e., PCA, IPCA, and PPCA, the appropriate number of principal components used to reconstruct the missing signal needs to be systematically quantified. In this paper, we utilize the VBPCA method to extract the CME from a regional GNSS position time series with missing values, and compare its performance with the other two approaches, PCA and IPCA. The PCA method is the most common algorithm with which to filter CME. The SOPAC uses this method to remove the CME in the PBO GNSS network. It can only handle a continuous residual coordinate matrix. Thus, it needs to fill the missing epoch using the spatially averaged values derived from other stations on these days. Then, traditional PCA analysis should be performed iteratively until convergence to refill in these missing points. In contrast, the IPCA algorithm can directly deal with an incomplete positional time series based on minimizing the weighted quadratic norm of principle component (PC) unknowns of an epoch. More details about PCA and IPCA algorithms can be found in Dong et al. [19] and Shen et al. [26], respectively. The formal errors of daily GNSS time series are not taken into account in this research, and its effect on CME extraction should be further explored. The remainder of this paper is organized as follows. Section 2 presents the VBPCA algorithm. Section 3 describes the data processing and demonstrates the effectiveness of this method by numerical synthetic and real time series analysis. Variations of the noise characteristics and the linear velocity uncertainty for the regional GNSS stations before and after removing CME are evaluated in Section 4; conclusions are summed up in Section 5.

## 2. Methodology

Since the GNSS measurement is often incomplete or missing in practice, the traditional approaches usually handle missing data by explicitly recovering them. The short-term missing data can be filled well by interpolation, yet long-term missing data are difficult to recover [26]. Moreover, most data interpolation methods focus only on single point time series, without regarding to the spatial correlation among points in a region [32]. This may result in incorrect extraction of CME. Here, we put forward the VBPCA algorithm to characterize and mitigate the impact of CME. The VBPCA method, including regularization, consideration of the noise term, and introduction of the a prior distribution over the model parameters, performs well in terms of finding the intrinsic dimension and the optimal number of clusters for the data with missing values [33].

### Variational Bayesian PCA

In the context of VBPCA, the GNSS observed datum $X=[x_{1},x_{2},\cdots ,x_{n}]\in R^{d\times n}$ is the stacking of all unfiltered residual time series $x_{j}$ that can be formulated as:

(1) $$x_{j}=Wp_{j}+\mu +e_{j},\;j=1,2,\cdots ,n$$

where d indicates the number of epochs, n is the number of GNSS stations, $W\in R^{d\times k}$ denotes the loading matrix, $p_{j}\in R^{k\times 1}$ are the latent variables or principal components, $\mu \in R^{d\times 1}$ is the bias vector, and $e_{i}\in R^{d\times 1}$ represents the noise term.

In addition to the assumption that the latent variables $p_{j}$ and noise $e_{j}$ are normally Gaussian distributed, the VBPCA also imposes a Gaussian a priori distribution on the parameters of μ and W to overcome the overfitting problem:

(2) $$\begin{array}{l} p(p_{j})=N(p_{j}|0,I)\\ p(e_{j})=N(e_{j}|0,\varepsilon_{x}I)\\ p(\mu )=N(\mu |0,\varepsilon_{\mu }I)\\ p(W)=\prod_{j=1}^{k}N(W_{:j}|0,\varepsilon_{W,j}I) \end{array}$$

where $\varepsilon_{x}$ is the noise variance, $\varepsilon_{\mu }$ and $\varepsilon_{W,j}$ represent the a priori variance for μ and $W_{:j}$ separately, $W_{:j}$ is the j-th column of the loading matrix W, and I denotes the Identity Matrices. In the VBPCA framework, $\varepsilon =\{\varepsilon_{x},\varepsilon_{\mu },\varepsilon_{W,j}\}$ is regarded as the hyperparameter set, and θ={W,p,μ} is treated as a hidden variable. Their values can be calculated by the variational expectation-maximum algorithm. Since the true posterior p(θ|x,ε) is analytically intractable, it is usually approximated by using a simple probabilistic density function q(θ). In this way, the calculation of the posterior p(θ|x,ε) is modified to update the approximation q(θ), so as to minimize the cost function in the E-step:

(3) $$C(q(\theta ),\varepsilon )=\int q(\theta )\log\frac{q(\theta )}{p(x,\theta |\varepsilon )}d\theta$$

Then the hyperparameters ε can be optimized with the obtained q(θ) to maximize the likelihood p(x|ε) in the M-step.

**Figure 1:**
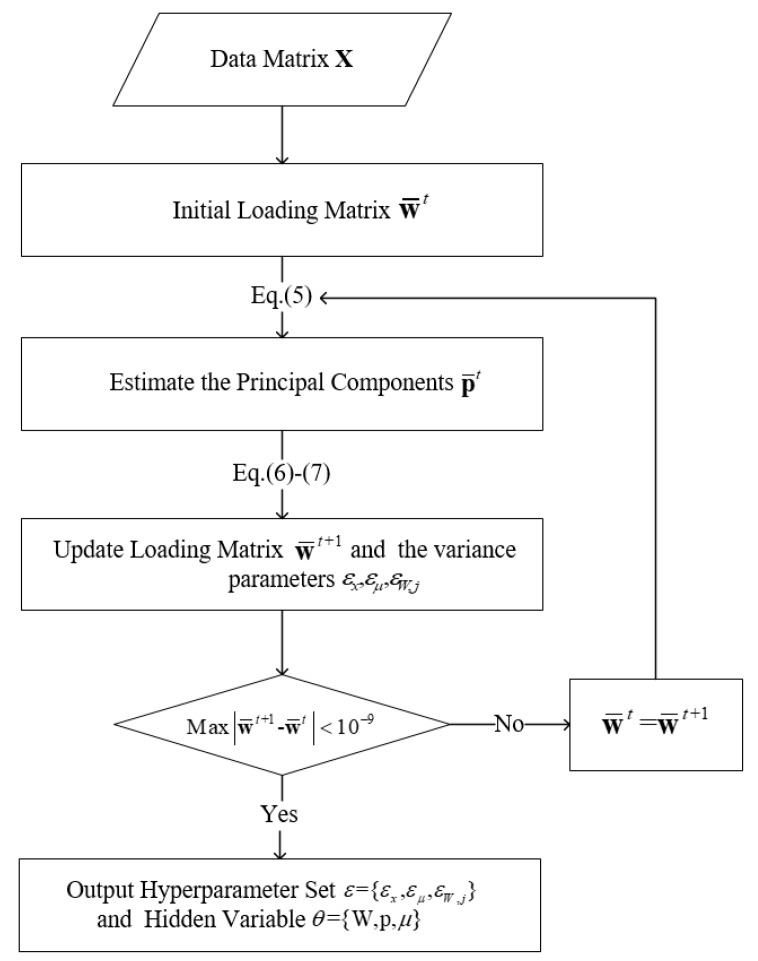
Flowchart of the VBPCA algorithm.

For the sake of calculation convenience, q(θ) is used in the following form:

(4) $$q(\theta )=\prod_{i=1}^{d}N(\mu_{i}|{\bar{\mu }}_{i},{\tilde{\mu }}_{i})\prod_{i=1}^{d}N(w_{i}|{\bar{w}}_{i},\Sigma_{w_{i}})\prod_{j=1}^{n}N(p_{j}|{\bar{p}}_{j},\Sigma_{p_{j}})$$

where $\mu_{i}$ is the i th element of the bias vector μ; $w_{i}$ is the column vector corresponding to the i th row of W; $p_{j}$ are the latent variables of the jth observations; and ${\bar{\mu }}_{i}$ and ${\tilde{\mu }}_{i}$ are the posterior mean and variance of μ. ${\bar{w}}_{i}$ and $\Sigma_{w_{i}}$ are the posterior mean and variance of $w_{i}$, and ${\bar{p}}_{j}$ and $\Sigma_{p_{j}}$ are the posterior mean and variance of $p_{j}$, respectively. Thus, the cost function (Equation (3)) can be minimized by update alternatively one factor of $q(\theta_{i})$, while other factors are held invariant. Finally, the model parameters and hyperparameters can be carried out by calculating the Equations (5)–(7) iteratively until convergence. The main steps and the corresponding flowchart of this algorithm (Figure 1) are as follows:

**Step1**: Update of the latent variables:

(5) $$\begin{array}{l} \Sigma_{p_{j}}=\varepsilon_{x}{\left(\varepsilon_{x}I+\sum_{i\in O_{j}}{\bar{w}}_{i}{\bar{w}}_{i}^{Τ}+\Sigma_{w_{i}}\right)}^{-1},\\ {\bar{p}}_{j}=\frac{1}{\varepsilon_{x}}\Sigma_{p_{j}}\sum_{i\in O_{j}}{\bar{w}}_{i}(x_{ij}-{\bar{\mu }}_{i}),\;j=1,\cdots ,n \end{array}$$

**Step2**: Estimation of the bias vector and loading matrix:

(6) $$\begin{array}{l} {\bar{\mu }}_{i}=\frac{\varepsilon_{\mu }}{\left|O_{i}\right|(\varepsilon_{\mu }+\frac{\varepsilon_{x}}{\left|O_{i}\right|})}\sum_{i\in O_{j}}\left[x_{ij}-{\bar{w}}_{{}_{i}}^{Τ}p_{j}\right],\\ {\tilde{\mu }}_{i}=\frac{\varepsilon_{x}\varepsilon_{\mu }}{\left|O_{i}\right|(\varepsilon_{\mu }+\frac{\varepsilon_{x}}{\left|O_{i}\right|})},\\ \Sigma_{w_{i}}=\varepsilon_{x}{\left(\varepsilon_{x}diag(\varepsilon_{W,i}^{-1})+\sum_{j\in O_{i}}\left[{\bar{p}}_{j}{\bar{p}}_{{}_{j}}^{Τ}+\Sigma_{p_{j}}\right]\right)}^{-1},\\ {\bar{w}}_{i}=\frac{1}{\varepsilon_{x}}\Sigma_{w_{i}}\sum_{j\in O_{i}}p_{j}(x_{ij}-{\bar{\mu }}_{i}],i=1,\cdots ,d \end{array}$$

where $x_{i,j}$ represents the element i,j of matrix X, O represents the set of indices i,j for which $x_{i,j}$ is observed, $O_{i}$ is the set of indices j for which $x_{i,j}$ is observed, and $\left|O_{i}\right|$ denotes the number of elements in $O_{i}$.

**Step3**: Calculation of variance parameters:

(7) $$\begin{array}{l} \varepsilon_{x}=\frac{1}{N}\sum_{ij\in O}\left[{\left(x_{ij}-{\bar{w}}_{i}^{Τ}{\bar{p}}_{j}-{\bar{\mu }}_{i}\right)}^{2}+{\tilde{\mu }}_{i}+{\bar{w}}_{i}^{Τ}\Sigma_{p_{j}}{\bar{w}}_{i}+{\bar{p}}_{j}^{Τ}\Sigma_{w_{i}}{\bar{p}}_{j}+tr(\Sigma_{p_{j}}\Sigma_{w_{i}})\right]\\ \varepsilon_{\mu }=\frac{1}{d}\sum_{i=1}^{d}({\bar{\mu }}_{i}^{2}+{\tilde{\mu }}_{i})\\ \varepsilon_{W,i}=\frac{1}{d}\sum_{i=1}^{d}({\bar{w}}_{ik}^{2}+{\tilde{w}}_{ik}) \end{array}$$

where ${\tilde{w}}_{ik}$ stands for the kth element on the diagonal of $\Sigma_{w_{i}}$.

The VBPCA can automatically select the optimal number of principal components for data reconstruct based on the hyperparameter $\varepsilon_{W,i}$, which tends to be zero if the evidence of the corresponding principal component for reliable data modeling is weak [30]. This characteristic is called the automatic relevance determination (ARD) and makes it easy to recover the missing values in the GNSS time series. Furthermore, the unreliable reconstruction of missing data can also be detected since the VBPCA method provides uncertainty information for unknown quantities.

## 3. Experiment and Analysis

### 3.1. GNSS Data Processing

**Figure 2:**
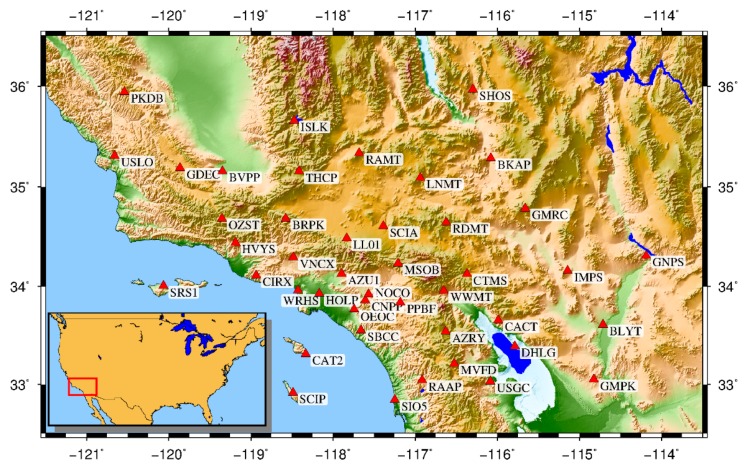
Geographical distribution of the selected 44 GNSS stations in Southern California.

We selected 44 continuous GNSS stations located in Southern California (Figure 2) to evaluate the VBPCA algorithm, and then compare its performance with the other two methods, including PCA and IPCA. The latest Scripps Orbit and Permanent Array Center (SOPAC) provides position time series spanning 14-years from 2005 to 2018 for the selected GNSS stations; they are used here during the experiment, so as to avoid the effects of mismodeling seasonal signals and noises on identifying CME [34]. Any position value with large formal error (>10, >10, >20 mm for the north, east, and up directions, respectively) is considered an outlier and first discarded from the raw time series. Then the linear trend, annual and semiannual terms, and the offsets are separately modeled and subtracted to obtain the residual position time series [35]. After that, the post-seismic displacements due to strong earthquakes are corrected, and the residuals that exceed three interquartile range (IQR) thresholds are also removed. Finally, we obtain the “clean” and unfiltered residual time series with an average missing rate of 5.25% for the experiment.

### 3.2. Comparison of CME Relative Errors from Different Methods

**Figure 3:**
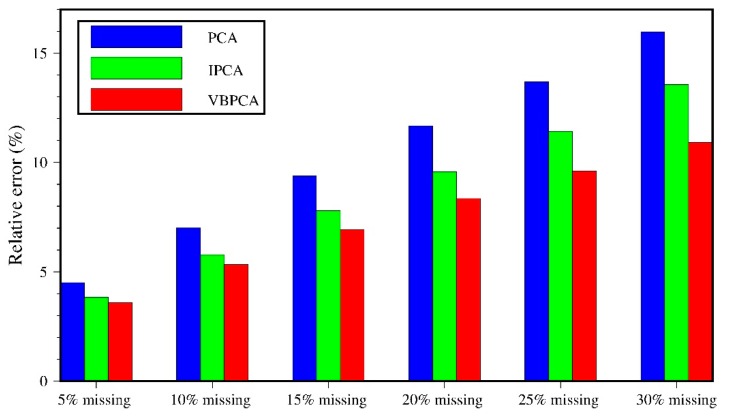
Relative errors of CME calculated by PCA (blue), IPCA (green), and VBPCA (red) with different percentages of deleted data.

We first filled the data gaps of the 44 GNSS residual time series to obtain fully complete coordinate matrix X using the regularized expectation-maximization (RegEM) algorithm [36]. Then we randomly picked up 5–30% observations with 5% increments and removed them from the time series to simulate the incomplete data. In this way, we can evaluate the capability of the VBPCA algorithm to extract CMEs with different amounts of missing data. Note that the gaps in the real GNSS observations are usually lost over a successive time span rather than a completely random absence [32]; thus, in the synthetic experiment, some of the deleted elements were distributed continuously, while the others were lost completely at random. Finally, we separately employed the PCA, IPCA, and VBPCA methods to extract the CMEs from our simulated complete and incomplete data. Since the CMEs for a regional GNSS network should be constant over a time span no matter whether the data are missing or not, we treat the CMEs from the simulated complete time series as the “true” reference for comparison purpose, and then calculate the relative errors of CME as [29]:

(8) $$\delta =\frac{1}{E}\sum_{k=1}^{E}\sqrt{\frac{\sum_{i=1}^{d}\sum_{j=1}^{n}{\left(CME_{i,j}^{k}-CME_{i,j}^{0}\right)}^{2}}{\sum_{i=1}^{d}\sum_{j=1}^{n}{\left(CME_{i,j}^{0}\right)}^{2}}}\times 100\%$$

where E which equals 100 here, and indicates the repeated number of every experiment; $CME_{}^{0}$ and $CME_{}^{k}$ are the common mode errors before and after introducing gaps, respectively. Figure 3 presents the average relative errors of CME calculated by PCA, IPCA, and VBPCA methods with different percentages of deleted data for the north component. We can see that the relative errors of CME computed by all the three methods become larger with increasing percentages of missing data. Within all experiments, the performance of VBPCA in estimating the CME is better than that of PCA and IPCA. When there is less missing data (i.e., 5%), the relative errors of CME calculated by the three methods are not very significant with the maximum distinction of less than 1%. However, with increasing missing data, the three results become much different. In particular, when 30% of the observations are removed from the time series, the relative errors of CME using VBPCA reach the maximum of only 10.9%, indicating improvements of 2.7% and 5.1% compared with IPCA (13.6%) and PCA (16.0%), respectively. Similar results were obtained for the east and up components. Therefore, we conclude that the VBPCA method is an alternative, effective way to obtain the CME from incomplete GNSS position time series, especially for larger number of stations with a bigger missing data rate.

### 3.3. The Performance of Missing Value Estimation

**Figure 4:**
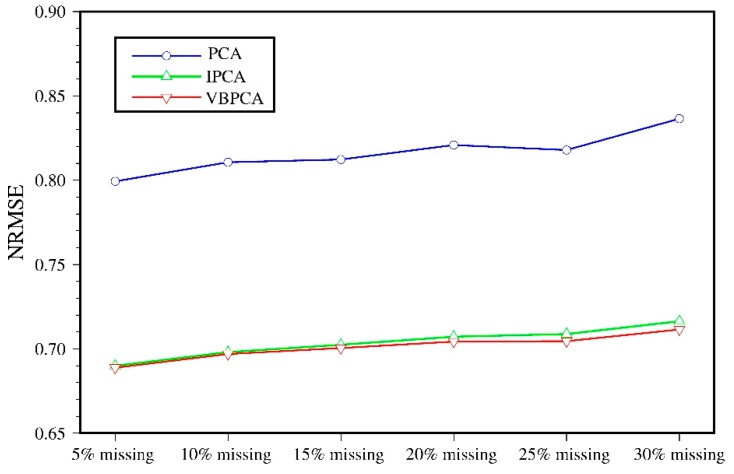
NRMSEs of PCA, IPCA, and VBPCA for various percentages of missing values.

An advanced feature of VBPCA is that it can automatically select the right number of principal components for missing data reconstruction. This property makes it convenient for GNSS missing value imputation. In order to evaluate the performance of missing value estimation, we selected twelve stations with missing data of less than 2% for artificial data deleting, and used the normalized root mean squared error (NRMSE) to assess the estimation accuracy. The introduced missing data are ranged from 5% to 30% of the total observation data with 5% increments, and the NRMSE is defined as:

(9) $$NRMSE=\frac{1}{E}\sum_{k=1}^{E}\sqrt{\frac{\frac{1}{N}{\left(x_{k}^{est}-x^{obs}\right)}^{2}}{var(x^{obs})}}$$

where E is the same as Equation (5), N is the number of deleted data, $x_{k}^{est}$ represents the estimation of the artificial deleted values, $x^{obs}$ indicates the real GNSS observed values corresponding to $x_{k}^{est}$, and var(·) defines the variance. The NRMSE value will come to 0 when the estimation is accurate, and 1 when the estimation is poor or the noise involved is too large. Figure 4 shows the NRMSE results of the three algorithms for the north component with various percentages of missing data. With rising missing data, the NRMSE values also present an increasing order for all the three methods, among which the VBPCA algorithm provides the most accurate results, followed by the IPCA method, while PCA exhibits the poorest. However, the difference between VBPCA and IPCA is not significant. Similar results are obtained for the east and up components; hence, we do not show the graphs here due to the limited space. Therefore, we conclude that the VBPCA method is more suitable to fill the missing values for GNSS time series, especially in the case of large amount of missing data.

## 4. Results and Discussion

### 4.1. Extraction of CME Using the VBPCA Method

**Figure 5:**
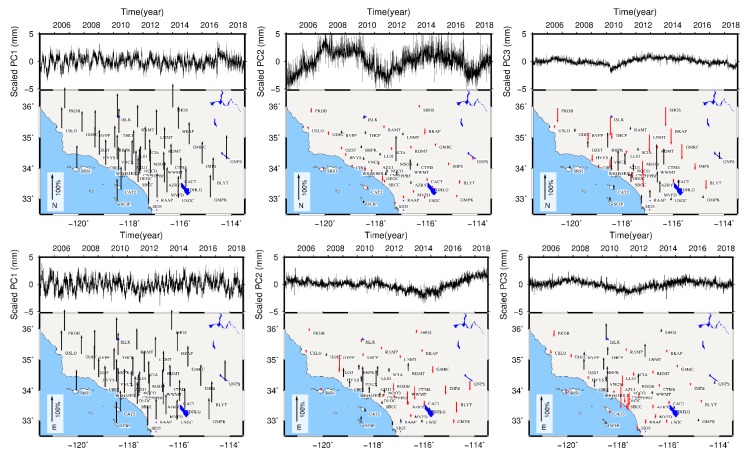

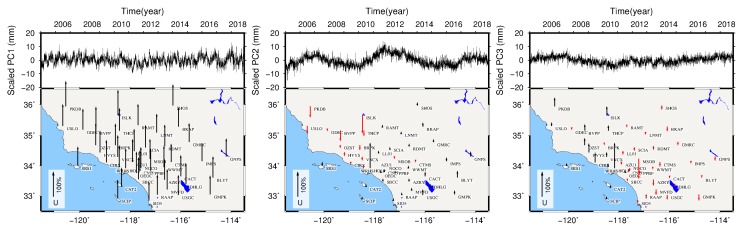
The first three scaled principal components (PCs: top) and their corresponding spatial responses (bottom) for the north (upper panels), east (middle panels), and up (lower panels) components of the 44 GNSS stations. The up-black and down-red arrows represent positive and negative responses to the scaled PCs, respectively.

Table 2, Table 3 and Table 4 show the a priori variance $\varepsilon_{W,j}$ corresponding to the first five principal components of the north, east, and up (NEU) components obtained by the VBPCA algorithm, respectively. We notice that the $\varepsilon_{W,1}$ values, which separately interpret 54.71%, 60.84%, and 54.84% of the total variance for the NEU components, are all significantly larger than the others. $\varepsilon_{W,2}$ contributes 10.00%, 6.43%, and 7.74% of the total variance, while the upper $\varepsilon_{W,j}$ accounts for no higher than 5.01% for each direction. We then scale the PCs/eigenvectors by the normalization factor similar to [19], and show the first three scaled PCs and its normalized eigenvectors of the VBPCA solution for the NEU components in Figure 5. It can be clearly seen that the normalized amplitudes of the first eigenvectors all have the same sign and show a positive spatial response for all the three components with uniform distribution. The mean responses are 89.32%, 83.34%, and 82.56% for the north, east, and up components, respectively, while the minimum values are 70.92% at station WWMT, 62.68% at station AZU1, and 71.32% at station GNPS. Quite different from the 1st PC, the 2nd and 3rd PCs show both positive and negative spatial responses in all directions, and the spatial patterns do not exhibit strong coherence over the network. In addition, some stations, e.g., MSOB in the north, WRHS in the east, and HOLP upward, show significantly larger responses compared with the other stations. We think that this may be due to local effects, unmodeled signals or errors, etc., which are uncommon for the entire regional stations. Therefore, in the following analysis, we will only use the 1st PC and its corresponding eigenvectors to recover the CME, and then subtract the CME from our regional GNSS residual time series to eliminate the spatial correlation of inter-stations.

### 4.2. Interstation Correlation Analysis

**Figure 6:**
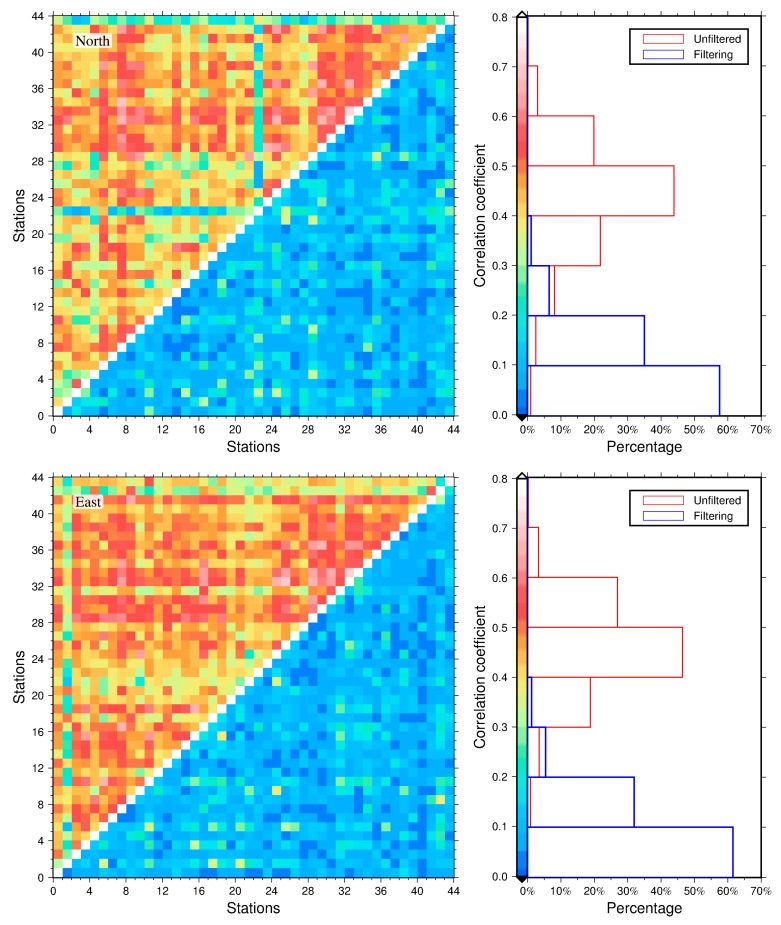

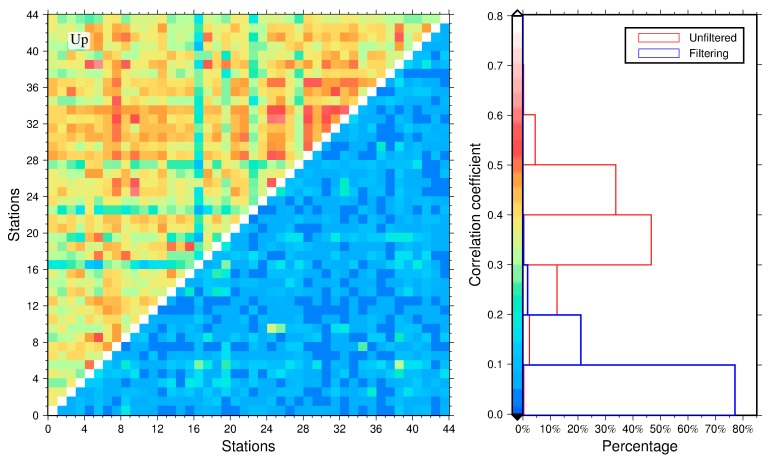
Interstation correlation coefficients (left panels) and the histograms (right panels) before and after filtering CME from residual time series for the north (top panels), east (middle panels), and up (bottom panels) components. The unfiltered and filtered interstation correlation coefficients are separately shown at the upper triangle and lower triangular of the left panels, while their corresponding histograms are shown as red and blue colors in the right panels, respectively.

The distance correlation coefficient is applied in this research to measure the dependency of two GNSS stations before and after reducing CME from the residual time series, due to the reason that this method can detect both linear and nonlinear correlation between two variables, whereas the classic Pearson’s correlation can only measure the linear relationship [37]. The values of distance correlate to the range between 0 and 1 (0 implies independence, 1 represents similarity). Figure 6 illustrates the interstation correlations among the 44 stations and their corresponding histograms before and after removing CME from the NEU residual time series. We notice that the 44 GNSS stations show a significant correlation before filtering, with average values of 0.43, 0.46, and 0.38 for the north, east, and up components, respectively, among which the maximum correlation reaches up to 0.71 between station SBCC and SCIA (about 120 km apart) for the east component. These results confirm that there are strong spatial correlations in the regional GNSS network, which need to be eliminated before scientific research and application. After removing CME with VBPCA, the average correlation coefficients are remarkably reduced to 0.11, 0.10, and 0.08, representing average interstation correlation reductions of 74.42%, 78.26%, and 78.95% for the NEU components, respectively. Hence, we conclude that the VBPCA filtering is an effective technique to reconstruct the CME for regional GNSS network.

### 4.3. Time Series Analysis

Table 5 lists the root mean square (RMS) values of the 44 GNSS residual time series before and after filtering CME with VBPCA for the NEU components, respectively. We can see that the RMS reduction ranges between 7.63% and 44.00% for all stations, with an average value of 27.11%, 28.15%, and 23.28% for the North, East, and Up components, respectively. In particular, station SBCC, PPBF and SCIA exhibits the maximum RMS reduction of 44.00% (1.15 vs. 0.64), 42.86% (1.20 vs. 0.69), and 33.35% (3.92 vs. 2.61) for the north, east, and up component.

**Figure 7:**
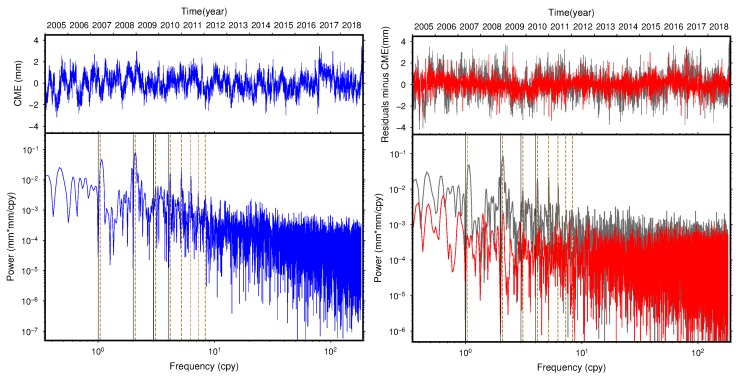

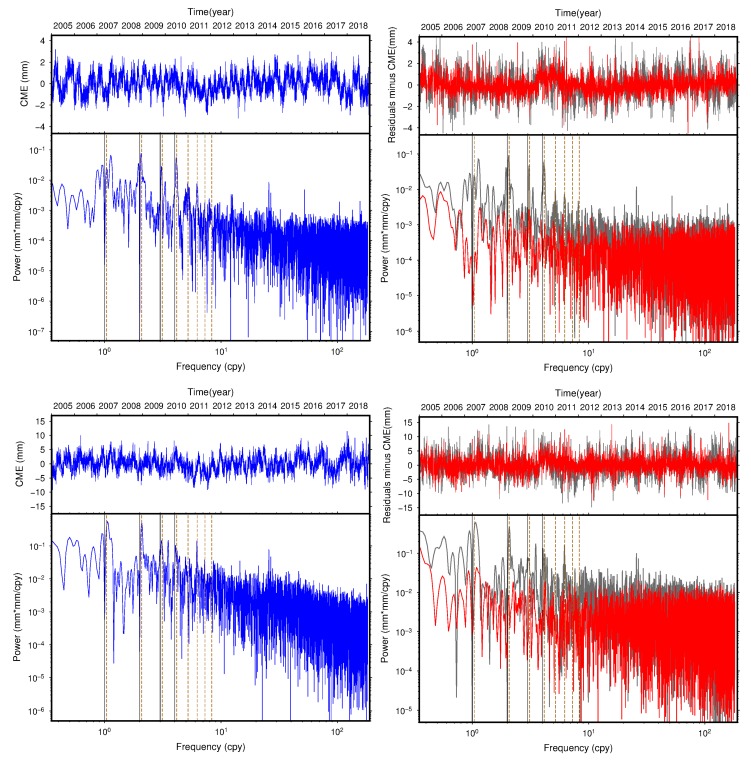
Power spectra of CME, unfiltered and filtered residual time series of station SCIA for the north (top panels), east (middle panels), and up (bottom panels) components. Left panels: CME and its power spectra (blue). Right panels: unfiltered (gray) and filtered time series (red), together with their corresponding power spectra. The vertical black-solid lines indicate harmonics of 1.0 cpy, and brown-dash lines indicate harmonics of 1.04 cpy.

To further analyze the periodicity of the residual time series, we take station SCIA as an example and calculate its power spectra using the Lomb–Scargle periodogram before and after removing CME for the NEU components. The CME and its power spectra are plotted in the left panels of Figure 7, while the unfiltered and filtered time series, together with their corresponding power spectra are shown in the right panels. It can be clearly seen that the 1.04 cycle per year (cpy), which is called the draconitic harmonic, shows high power with significant peaks for all the three components in the CME power spectra, and the noise characteristics of the CME are proximate to pure flicker noise. The spectrum of the unfiltered time series shows a combination of colored noise at low frequencies and white noise at high frequencies for all three directions, while there is a clear decrease in the power spectra at low frequencies compared with that of the high frequencies, especially for several peaks of the draconitic harmonic frequencies after eliminating CME with VBPCA. This indicates that the CME of the selected region contains part of flicker noise and draconitic signals, which can be significantly reduced with VBPCA filtering.

### 4.4. Effect of CME on Noise Amplitude and Velocity Estimation

**Figure 8:**
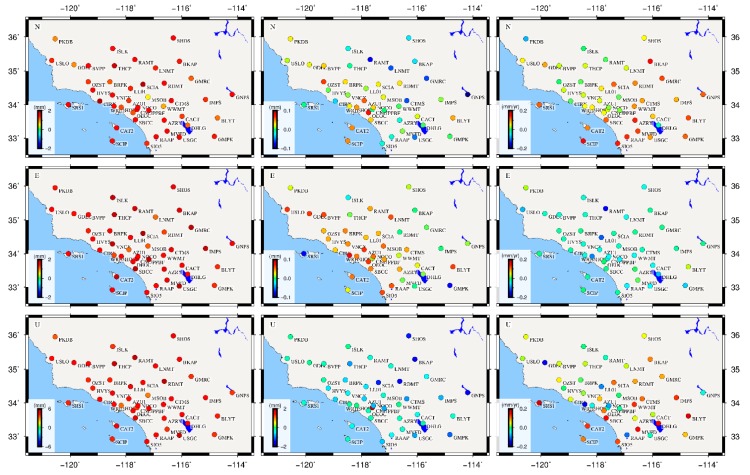
The variation of amplitudes of PLN (left panels) and WN (middle panels) before and after CME filtering with VBPCA, together with the velocity differences (right panels). From top to bottom panels represent the North, East and Up components.

**Figure 9:**
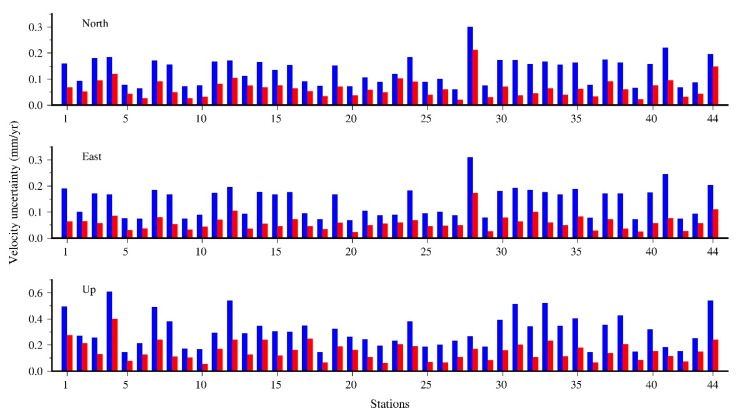
Velocity uncertainty of the 44 GNSS stations for the north, east, and up (NEU) components before (blue bars) and after (red bars) CME filtering with VBPCA.

To quantitatively analyze the effects of CME filtering on the final velocity field estimation, we estimate the velocity together with the PLN+WN noise model using Hector software [38] for the total 44 stations. Figure 8 illustrates the variation (unfiltered minus filtered) of the amplitudes of PLN and WN, and the velocity, after removing CME by VBPCA algorithm for the NEU components. Figure 9 presents the corresponding velocity uncertainty before and after CME filtering. We observe that the PLN amplitudes exhibit a significant decrease after filtering out CME, with average values of 49.90%, 54.56%, and 49.75% in the NEU directions, respectively. However, the WN amplitudes only vary with average decrease ratios of 1.8% and 5.82% for the north and east components, but an increase ratio of 27.65% for the up component. This is due to the fact that flicker noise is dominated in the CME and can be remarkably suppressed using VBPCA filtering, while white noise is likely related to site-specific errors that cannot be removed by CME filtering.

The velocity differences vary between −0.27 mm/year and 0.16 mm/year for the horizontal components, and −0.36–0.21 mm/year for the up component (right panels of Figure 8), indicating that CME has a certain influence on the velocity estimation of the GNSS stations. Compared with velocity difference, the velocity uncertainties decline obviously with averages of 51.95%, 57.31%, and 49.92% after filtering out CME for the NEU components, among which the maximum improvement ratio equals 79.07% at station SRS1 for the east component, while the minimum improvement is 12.02% at station MSOB for the up component. Therefore, we conclude that CME filtering would be essential to improve the precision of velocity estimates.

## 5. Conclusions

In this paper, we introduce an alternative VBPCA approach to extract CME from regional GNSS position time series with missing values. Without establishing the covariance matrix and computing eigenvalue decomposition, the VBPCA method considers PCA from a probabilistic point of view, and utilizes the variational expectation-maximization iterative algorithm to search principal subspace. Moreover, The VBPCA can automatically select the optimal number of principal components for data reconstruction based on the hyperparameter $\varepsilon_{W,i}$, which makes it convenient to recover the missing values in the GNSS time series.

The daily position time series of 44 continuous GNSS stations located in Southern California from SOPAC are selected to simulate and investigate the performance of filtering out CME with VBPCA. Our results show that the VBPCA algorithm achieves lower relative errors of CME compared with the other two widely used approaches; namely, PCA and IPCA. In particular, it shows maximum improvements of 2.7% and 5.1% compared to IPCA and PCA in the case of 30% missing data. With respect to the performance of missing value estimation, the VBPCA algorithm always exhibits the lowest NRMSE; thus, it is more suitable for filling the gaps of the GNSS time series, especially for a large amount of missing data.

We then apply the VBPCA algorithm to the 44 incomplete residual time series, and successfully recover the CME using the first PC and its eigenvector for the selected regional GNSS network. After removing CME, both the interstation correlation coefficients and the RMS values of the residual time series show remarkable decreases. The draconitic harmonics, especially for the top eight frequencies, also exhibit significant reductions. Therefore, we conclude that the CME indeed exists in the regional GNSS network and the VBPCA method performs quite well to reconstruct the CME for the selected region.

Meanwhile, the velocities of unfiltered and filtered position time series, together with their corresponding velocity uncertainties are simultaneously estimated with the PLN+WN noise model. We observe that after CME filtering, the PLN amplitudes are significantly reduced, with average values of 49.90%, 54.56%, and 49.75% in the NEU directions, respectively. However, the WN amplitudes exhibit only slight decreases in their horizontal components, but increases in the up direction. The velocity differences can reach 0.36 mm/year, indicating that CME has a certain influence on the velocity estimation of the GNSS stations. On the other hand, the velocity uncertainty represents an average reduction of 51.95%, 57.31%, and 49.92% for the three components; thus, we conclude that the CME filtering with VBPCA could not only attenuate the colored noise, but also improve the accuracy of the estimated station velocity. Further work is still required to take the formal errors into account during the VBPCA implementation, which may also have some effect on the CME extraction.

Finally, with the fast expansion of GNSS stations and the accumulated longer-time, or higher-rate GNSS observations, we are facing a challenge to find an optimal and objective approach to extract CME for regional GNSS network. The deep leaning method that is regarded as a learning technique on artificial neural networks can provide fast and effective solutions, especially in the analysis of big data. Considering the advantages of deep learning, its application in extracting the CME from GNSS position time series will be further explored.

## Figures and Tables

**Table 1:** Advantages and disadvantages of various mathematical methods used so far to filter common mode error (CME).

Filtering Methods	Advantages	Disadvantages
Stacking	Simple to calculate Ability to handle missing data	Not suitable for larger scale GNSS networks Inability to identify stations with strong local effects Need to determine realistic weight values
Reference frame transformation	Simple to calculate Ability to handle missing data	Distorted signal or residual CME may appear at the edge of the region GNSS positions cannot reveal movement from the global point of view
Statistical signal decomposition techniques	Rigorous mathematical framework Ability to identify stations with strong local effects Suitable for larger scale GNSS networks	Inability to handle missing data for the traditional methods Any two time series need common epochs for the modified methods

**Table 2:** The first five $\varepsilon_{W,j}$ obtained by VBPCA algorithm for the north component.

Principal Component	$\varepsilon_{W,j}$	Difference	Proportion (%)	Histogram
1	0.78	0.64	54.71	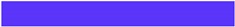
2	0.14	0.07	10.00	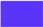
3	0.07	0.01	5.01	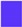
4	0.06	0.01	4.02	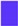
5	0.05	-	3.48	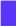

**Table 3:** The first five $\varepsilon_{W,j}$ obtained by VBPCA algorithm for the east component.

Principal Component	$\varepsilon_{W,j}$	Difference	Proportion (%)	Histogram
1	0.94	0.84	60.84	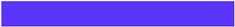
2	0.10	0.04	6.43	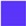
3	0.06	0.01	3.97	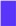
4	0.06	0.01	3.59	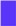
5	0.05	-	3.00	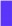

**Table 4:** The first five $\varepsilon_{W,j}$ obtained by VBPCA algorithm for the up component.

Principal Component	$\varepsilon_{W,j}$	Difference	Proportion (%)	Histogram
1	7.29	6.26	54.84	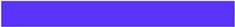
2	1.03	0.42	7.74	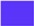
3	0.61	0.17	4.60	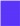
4	0.44	0.05	3.33	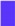
5	0.39	-	2.96	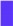

**Table 5:** RMS values of the 44 GNSS residual time series before and after removing CME (unit: mm).

No.	Station	Unfiltered			Filtered			No.	Station	Unfiltered			Filtered		
		N	E	U	N	E	U			N	E	U	N	E	U
1	AZRY	1.23	1.44	4.27	0.94	1.08	3.36	23	MSOB	2.42	1.63	5.36	2.24	1.26	4.74
2	AZU1	1.45	1.55	4.96	1.09	1.36	3.96	24	MVFD	1.41	1.54	4.55	1.01	1.23	3.70
3	BKAP	1.39	1.30	4.18	1.09	0.93	3.35	25	NOCO	1.27	1.41	3.99	0.84	1.01	2.91
4	BLYT	1.36	1.38	4.65	1.04	1.01	3.77	26	OEOC	1.48	1.32	3.93	1.10	0.88	2.82
5	BRPK	1.42	1.43	4.12	1.15	1.02	3.04	27	OZST	1.31	1.62	4.61	1.04	1.15	3.56
6	BVPP	1.13	1.65	4.72	0.72	1.17	3.56	28	PKDB	1.41	1.44	5.79	1.16	1.07	4.78
7	CACT	1.21	1.36	4.25	0.88	0.96	3.23	29	PPBF	1.31	1.20	3.85	0.91	0.69	2.59
8	CAT2	1.11	1.22	3.40	0.67	0.76	2.35	30	RAAP	1.18	1.22	3.84	0.71	0.77	2.86
9	CIRX	1.32	1.35	4.28	0.88	0.92	3.13	31	RAMT	1.14	1.26	3.78	0.74	0.81	2.61
10	CNPP	1.27	1.47	4.09	0.86	1.12	3.20	32	RDMT	1.15	1.50	3.97	0.80	1.19	2.82
11	CTMS	1.28	1.41	3.91	1.00	0.99	3.11	33	SBCC	1.15	1.24	3.96	0.64	0.72	2.73
12	DHLG	1.22	1.49	4.36	0.98	1.16	3.41	34	SCIA	1.11	1.24	3.92	0.65	0.77	2.61
13	GDEC	1.33	1.18	3.79	1.07	0.83	2.72	35	SCIP	1.21	1.44	4.06	0.84	1.05	3.23
14	GMPK	1.19	1.42	4.10	0.82	1.04	3.29	36	SHOS	1.31	1.28	4.14	0.94	0.88	3.10
15	GMRC	1.52	1.33	4.14	1.26	0.93	3.34	37	SIO5	1.19	1.19	3.49	0.79	0.76	2.50
16	GNPS	1.31	1.34	3.92	0.93	0.93	3.13	38	SRS1	1.25	1.34	4.07	0.88	0.96	3.26
17	HOLP	1.41	1.47	6.48	1.13	0.97	5.97	39	THCP	1.22	1.38	4.03	0.77	0.92	2.78
18	HVYS	1.37	1.33	4.39	0.97	1.00	3.35	40	USGC	1.26	1.39	4.44	0.93	1.05	3.62
19	IMPS	1.20	1.35	3.76	0.86	0.95	2.91	41	USLO	1.29	1.50	4.69	1.01	1.19	3.75
20	ISLK	1.51	1.75	5.98	1.22	1.43	5.00	42	VNCX	1.34	1.33	4.21	0.94	0.84	3.13
21	LL01	1.43	1.42	4.38	1.11	1.11	3.30	43	WRHS	1.23	1.99	4.23	0.87	1.66	3.16
22	LNMT	1.46	1.59	3.62	1.09	1.30	2.67	44	WWMT	1.75	1.77	5.10	1.61	1.44	4.28

## Nomenclature

CME	common mode error
GNSS	global navigation satellite systems
VBPCA	variational Bayesian principal component analysis
PC	principal component
NEU	north, east, and up
RMS	root mean square
WN	white noise
PLN	power-law noises
MLE	maximum likelihood estimation
PCA	principal component analysis
IPCA	improved principal component analysis
PPCA	probabilistic principal component analysis
VBPCA	variational Bayesian principal component analysis
MSSA	multi-channel singular spectrum analysis
ICA	independent component analysis
SOPAC	SCRIPPS ORBIT AND PERMANENT ARRAY CENTER
IQR	interquartile range
ARD	automatic relevance determination
RegEM	regularized expectation-maximization
NRMSE	normalized root mean squared error
cpy	cycle per year
d	the number of epochs
n	the number of GNSS stations
k	the number of principal components
X	d×n data matrix
$x_{j}$	*j*-th column of X
W	d×k loading matrix
$W_{:j}$	*j*-th column of the loading matrix W
$w_{i}$	the column vector corresponding to the i th row of W
${\bar{w}}_{i}$	posterior mean of $w_{i}$
$\Sigma_{w_{i}}$	posterior covariance of $w_{i}$
${\tilde{w}}_{ik}$	the k-th element on the diagonal of $\Sigma_{w_{i}}$
P	k×n matrix of principal components
$p_{j}$	*j*-th column of P
${\bar{p}}_{j}$	posterior mean of $p_{j}$
$\Sigma_{p_{j}}$	posterior covariance of $p_{j}$
μ	the bias vector
${\bar{\mu }}_{i}$	posterior mean of μ
${\tilde{\mu }}_{i}$	posterior variance of μ
$e_{j}$	*j*-th out of *n* noise vector
$\varepsilon_{x}$	the noise variance
$\varepsilon_{\mu }$	priori variance of μ
$\varepsilon_{W,j}$	priori variance of $W_{:j}$
ε	the hyperparameter set ($\varepsilon =\{\varepsilon_{x},\varepsilon_{\mu },\varepsilon_{W,j}\}$)
I	the Identity Matrices
θ	the hidden variable (θ={W,p,μ})
*C*	cost function to be minimized
O	the set of indices i,j for which $x_{i,j}$ is observed
$O_{i}$	the set of indices j for which $x_{i,j}$ is observed
$\left|O_{i}\right|$	the number of elements in $O_{i}$
N(x|μ,ε)	Gaussian normal probability density function over variable x with mean μ and covariance ε
